# Calcitonin gene‐related peptide inhibits angiotensin II‐induced NADPH oxidase‐dependent ROS via the Src/STAT3 signalling pathway

**DOI:** 10.1111/jcmm.15288

**Published:** 2020-05-05

**Authors:** Hong‐min Luo, Xia Wu, Xian Xian, Lu‐yao Wang, Liang‐yu Zhu, Hong‐yu Sun, Lei Yang, Wen‐xuan Liu

**Affiliations:** ^1^ Department of Nephrology Third Hospital Hebei Medical University Shijiazhuang China; ^2^ The Third Hospital Hebei Medical University Shijiazhuang China; ^3^ Department of Epidemiology and Statistics School of Public Health Hebei Key Laboratory of Environment and Human Health Hebei Medical University Shijiazhuang China

**Keywords:** Ang II, CGRP, ROS, Src, STAT3, VSMCs

## Abstract

We had previously demonstrated that the calcitonin gene‐related peptide (CGRP) suppresses the oxidative stress and vascular smooth muscle cell (VSMC) proliferation induced by vascular injury. A recent study also indicated that CGRP protects against the onset and development of angiotensin II (Ang II)‐induced hypertension, vascular hypertrophy and oxidative stress. However, the mechanism behind the effects of CGRP on Ang II‐induced oxidative stress is unclear. CGRP significantly suppressed the level of reactive oxygen species (ROS) generated by NADPH oxidase in Ang II‐induced VSMCs. The Ang II‐stimulated activation of both Src and the downstream transcription factor, STAT3, was abrogated by CGRP. However, the antioxidative effect of CGRP was lost following the expression of constitutively activated Src or STAT3. Pre‐treatment with H‐89 or CGRP_8–37_ also blocked the CGRP inhibitory effects against Ang II‐induced oxidative stress. Additionally, both in vitro and in vivo analyses show that CGRP treatment inhibited Ang II‐induced VSMC proliferation and hypertrophy, accompanied by a reduction in ROS generation. Collectively, these results demonstrate that CGRP exhibits its antioxidative effect by blocking the Src/STAT3 signalling pathway that is associated with Ang II‐induced VSMC hypertrophy and hyperplasia.

## 1 INTRODUCTION

Accumulating evidence has shown that the proliferation and migration of vascular smooth muscle cells (VSMCs) contribute greatly to the development of atherosclerosis.^1^, ^2^ Angiotensin II (Ang II), the primary effector molecule of the renin‐angiotensin system, plays a major role in the regulation of vascular function and structure. Ang II can increase arterial pressure and induce VSMC hypertrophy, proliferation and migration.^3^ It mediates its pathophysiological effects on VSMCs through multiple intracellular signalling pathways. Several studies have indicated that reactive oxygen species (ROS), which participate in the pathogenesis of cardiovascular diseases, are involved in mediating the signal transduction of Ang II‐induced hypertrophy.^4^ All vascular cell types, including endothelial cells and VSMCs, are capable of producing ROS. Ang II‐stimulated ROS are generated in VSMCs through the activity of NADPH oxidase, which contains five components (p47phox, p67phox, p40phox, p22phox and gp91phox).^5^ During this Ang II‐mediated activation of the enzyme, the phosphorylation of p47phox, in particular, is critical, as it triggers the formation of a complex between p47phox and ROS production–related molecules in the cytoplasm, enhancing their translocation to the membrane.^6^

The calcitonin gene‐related peptide (CGRP), which is composed of 37 amino acids and produced by alternative splicing of the primary transcript of the calcitonin/*CGRP* gene, is a potent vasodilator and hypotensive peptide.^7^ CGRP elicits its biological actions via non‐selective interaction with the calcitonin receptor‐like receptor (CRLR), receptor activity‐modifying protein 1 (RAMP1) and the intracellular receptor component protein (RCP), following the activation of the cAMP/protein kinase A (PKA)–dependent pathway.^8^ CGRP has been shown to exert various effects within the cardiovascular system, including the abrogation of antioxidative stress and inhibition of VSMC proliferation and migration.^9^ Recently, we reported that endogenous CGRP suppresses VSMC proliferation and oxidative stress induced by vascular injury.^10^ In addition, CGRP protects against the onset and development of Ang II‐induced hypertension, vascular hypertrophy and oxidative stress.^11^ These data confirmed the hypothesis that CGRP plays a protective role against Ang II‐induced oxidative stress in VSMCs, and the antioxidative effect may be associated with the pathophysiological effects on these muscle cells. However, the underlying cellular mechanisms involved are still unclear, and the critical CGRP‐regulated signal transduction pathways are yet to be identified.

Ang II binding to the signalling molecules that modulate ROS production in vascular cells is a complex mechanism that may occur at the transcriptional or post‐transcriptional level, involving the generation of several intermediate signalling molecules.^4^ Src, belonging to the family of non‐receptor tyrosine kinases, is one such signalling molecule. Src regulates several cellular pathways by phosphorylating the proteins involved in the mechanical and chemical stimulation processes that modulate ROS generation in VSMCs^12^ or regulate VSMC proliferation and migration in response to interaction with Ang II.^13^ Several studies have revealed numerous intracellular events occurring from the point of Src kinase activation to the expression of downstream target proteins, such as the signal transducer and activator of transcription 3 (STAT3).^14^, ^15^, ^16^ Src mediates the activation of STAT3, which subsequently regulates various VSMC processes, including cell proliferation and migration.^17^ Additionally, the roles of STAT3 in regulating ROS production and oxidative metabolism have recently been highlighted.^18^ However, very little is known about the association between Ang II and the Src/STAT3 signalling pathway modulating ROS production in VSMCs. As adrenomedullin, a member of the calcitonin peptide superfamily, inhibits Ang II‐induced oxidative stress via the Csk‐mediated inhibition of Src activity,^19^ we have been suggested that the Src/STAT3 signalling pathway would be a potential pathway through which CGRP interrupts Ang II‐induced ROS production. Therefore, in this study, we investigated whether the Src/STAT3 signalling pathway is associated with the antioxidative effect of CGRP and whether it consequently prevents Ang II‐induced hypertrophy and hyperplasia of VSMCs in vitro and in vivo.

## 2 MATERIALS AND METHODS

### 2.1 Materials

Human CGRP, human CGRP_8–37_, Ang II, N‐acetyl‐L cysteine (NAC), apocynin, PP2, H‐89 and dibutyl‐cAMP were purchased from Sigma. Niclosamide was purchased from Selleck Chemicals; anti‐phospho‐Src (Tyr416), anti‐phospho‐STAT3 (Tyr705), Src and STAT3 antibodies, from Cell Signaling Technology Inc; antibodies for p47phox and GAPDH, and all secondary antibodies, from Santa Cruz Biotechnology; antibodies of RAMP1, CRLR and ATP1a1, from Santa Cruz Biotechnology; RCP antibodies, from NOVUS Institute of Biotechnology; Alzet mini‐osmotic pumps (Alzet model 1004), from DURECT Corp; and the Src (B0107) and STAT3 (F0806) ORF cDNA clones, from GeneCopoeia. Other chemicals and reagents were of analytical grade.

### 2.2 Animals

Two‐month‐old male C57BL/6J mice, weighing 18‐25 g, and 80‐100 g male Sprague‐Dawley rats were obtained from the Beijing Vital River Laboratory Animal Technology Co. Ltd. All experiments were performed according to the Guidelines for the Care and Use of Laboratory Animals. The experiments were approved by the Local Committee on Animal Care, Use and Protection of Hebei Medical University. C57BL/6 mice were randomly assigned to 3 groups for treatment: infusion with normal saline (control) (n = 7), Ang II (750 µg/kg/d; in saline) (n = 7) and Ang II plus CGRP (300 ng/kg/h) (n = 7). Ang II and CGRP were put into separate pumps and administered subcutaneously in saline via an Alzet mini‐osmotic pump for 14 days.

### 2.3 VSMCs

Rat VSMCs were isolated from the aorta of 80‐100 g male Sprague‐Dawley rats and cut into small pieces as previously described.^10^ The tunica medium was separated from the adventitia and endothelium and cultured in DMEM (Invitrogen) supplemented with 10% FBS and a mixture of 100 U/mL penicillin and 100 μg/mL streptomycin (Invitrogen). α‐actin testing of cultured cells confirmed a positive response. The VSMCs were maintained at 37°C in a humidified atmosphere containing 5% CO_2_, and only passage 3 to passage 5 cells at 70%‐80% confluence were used in the experiments, except if stated otherwise. Each individual experiment was repeated at least thrice with different cell preparations.

### 2.4 Histology and immunohistochemistry

The abdominal aortas were excised from each mouse, fixed in 4% paraformaldehyde for 24 hours and embedded in paraffin. The aortas were then cut into 5‐μm sections, which were stained with haematoxylin and eosin (HE) and Elastica Van Gieson (EVG). For immunohistochemical analysis, arterial sections were incubated with rabbit anti‐8‐OHdG (1:100, Bioss, catalogue # bs‐1278R). The analysis software BZ‐II analyzer (Keyence) was applied to acquire the immunofluorescence average optical density (AOD) for evaluating the expression level of p‐Src and p‐STAT3 in VSMCs.

### 2.5 Immunofluorescence assay

VSMCs were fixed in 4% paraformaldehyde solution for 15 minutes at actual temperature, washed with PBS thrice and incubated in 5% normal goat serum blocking solution for 30 minutes in a humidified chamber at room temperature. Cells were incubated in anti‐p‐Src (1:25), and anti‐p‐STAT3 (1:25) for 12 hours at room temperature, washed thrice with PBS and incubated in fluorescein‐conjugated secondary antibodies for 60 minutes at room temperature. The cells were then washed with PBS, mounted with DAPI for 5 minutes and visualized using laser scanning confocal microscope. The analysis software BZ‐II analyzer (Keyence) was applied to acquire the immunofluorescence average optical density (AOD) for evaluating the expression level of p‐Src and p‐STAT3 in VSMCs.

### 2.6 Reactive oxygen species assay

Intracellular ROS was measured using DCFH‐DA fluorescent dye (Beyotime) according to the manufacturer's instructions. Briefly, cells were pre‐treated with or without test compounds for the indicated time periods and then stimulated with or without Ang II (10^−7^ mol/L) for 30 minutes and CGRP (10^−6^‐10^−8^mol/L) for 60 minutes. In some experiments, CGRP_8–37_ or H‐89 was added 30 minutes, dibutyl‐cAMP 60 minutes and apocynin (10^−6^ mol/L) 2 hours before CGRP treatment. The cells were then incubated with 10 µmol/L DCFH‐DA diluted in serum‐free culture medium for 20 minutes at 37°C in the dark. After incubation, the cells were washed twice with PBS and measured at 488‐nm excitation and 520‐nm emission using a microplate reader.

### 2.7 NADP/NADPH assay

Quantitation of the intracellular NADP/NADPH ratio was measured using the Amplite Fluorimetric NADP/NADPH assay kit (AAT Bioquest, Inc), according to the manufacturer's instructions. Briefly, cells were pre‐treated with or without test compounds for the indicated time periods and then stimulated with or without Ang II (10^−7^ mol/L) for 30 minutes and CGRP (10^−6^‐10^−8^mol/L) for 60 minutes. In some experiments, CGRP_8–37_ or H‐89 was added 30 minutes, dibutyl‐cAMP 60 minutes and apocynin (10^−6^ mol/L) 2 hours before CGRP treatment. NADP/NADPH ratio measurements were determined based on an enzymatic cycling reaction, using a Microplate reader (excitation: 540 nm and emission: 590 nm).

### 2.8 cAMP and PKA measurement

VSMCs were first pre‐treated Ang II (10^−7^ mol/L) for 30 minutes and then stimulated with or without and CGRP (10^−7^ mol/L) for 60 minutes and CGRP_8–37_ 30 minutes. cAMP and PKA levels of the VSMCs were measured using parameter cAMP assay (R&D Systems) and protein kinase A (PKA) ELISA (Mlbio) kits, respectively, according to the manufacturers’ protocols.

### 2.9 Transient transfection

For the transfection, VSMCs were cultured on a 6‐well plate at a density of 1 × 10^5^ cells/well for 24 hours. Then, the cells were transfected with 150 ng of Src ORF cDNA, STAT3 ORF cDNA or blank pReceiver‐M13 vector (control) for 48 hours, and the transfection efficiency was confirmed via immunoblotting for total protein.

### 2.10 Western blot analysis

Cell lysates were collected by means of immunoblotting as previously described. In brief, VSMCs, stimulated with for CGRP (10^−7^mol/L) for 60 minutes, and CGRP _8‐37_ (3 × 10^−5^mol/L) for 30 minutes, were treated with Ang II (10^−7^ mol/L) 30 minutes. In some experiments, dibutyl‐cAMP (10^−3^ mol/L) was applied for 60 minutes, or H‐89 (10^−5^ mol/L) for 30 minutes, PP2 (10^−5^ mol/L) for 20 minutes and niclosamide (10^−5^ mol/L) for 20 minutes before the CGRP pre‐treatment. After appropriate treatments, VSMCs extracts containing equal amounts of total protein were resolved by 10% SDS‐PAGE, and electro‐transferred to a PVDF membrane. After blocking in 5% skim milk, the membranes were incubated with primary antibodies (1:1000—RAMP1, PCR, p‐Src, p‐STAT‐3, STAT‐3, Src and ATP1a1; and 1:2000—GAPDH) followed by the appropriate secondary antibodies. For quantification, Western blot images were captured and analysed using Scion Image.

To determine p47phox translocation, the membrane and cytosolic fraction were separated by ultracentrifugation. p47phox (1:100) abundance was also assessed in membrane and cytoplasmic fractions.

### 2.11 Quantitative real‐time PCR

After appropriate treatments, total RNA from VSMCs was extracted using TRIzol reagent (Invitrogen) according to the manufacturer's protocol. The cDNA was synthesized using reverse transcriptase (Invitrogen). Real‐time PCR was carried out using an Applied Biosystems 7500 fast PCR System (Life Technologies, USA) and SYBR Green RT‐PCR Kit (Invitrogen). Values were normalized to rat GAPDH. Oligonucleotide primers for real‐time PCR amplification (Table 1) were synthesized by Sangon Biotech (Shanghai).

**TABLE 1:** The primers used for real time RT‐PCR

Gene name	5′‐3′ sequence	Size
Rat GAPDH		
Forward	ACTCCCATTCTTCCACCTTTG	105 bp
Reverse	CCCTGTTGCTGTAGCCATATT	
Rat RAMP1		
Forward	GCTGGCTCATCATCTCTTCAT	107 bp
Reverse	CTATGGTCTCCATGTCCTCTTTG	
Rat CLR		
Forward	GCTGAGGAGGTGTATGACTATG	99 bp
Reverse	TGCTTGAACCTCTCCGTTAAA	
Rat RCP		
Forward	GCTCAGTAACTACGAGGTGTTC	112 bp
Reverse	GTAGGTGATGGCGTTCAAGT	
Rat p47phox		
Forward	AGACCCTGAGCCCAACTATG	181 bp
Reverse	CAGGTACATGGACGGGAAGT	

### 2.12 Cell proliferation assays

VSMCs (1 × 10^5^) were cultured in 96‐well plates, incubated in DMEM (Invitrogen) without serum for 16 hours and then treated with CGRP, Ang II or NAC for 12 hours. VSMC proliferation was then measured based on 5‐bromo‐2′‐deoxyuridine (BrdU) uptake using a Cell Proliferation ELISA Kit (Millipore Cat. No. 2750) according to the manufacturer's specifications.

### 2.13 Blood pressure evaluation

Systolic blood pressure was measured on days 0 (basal), 7 and 14 before pump implantation and every 3‐6 days after implantation using a standard tail cuff (BP‐2010A System, Softron).

### 2.14 Statistical analysis

Values are expressed as means ± SEM. Student's t test was used to determine significant differences between two groups. One‐way ANOVA was used to determine significant differences between three or more groups. Values of *P* < .05 were considered significant.

## 3 RESULTS

### 3.1 CGRP inhibited oxidative stress in Ang II‐induced VSMCs

**FIGURE 1:**
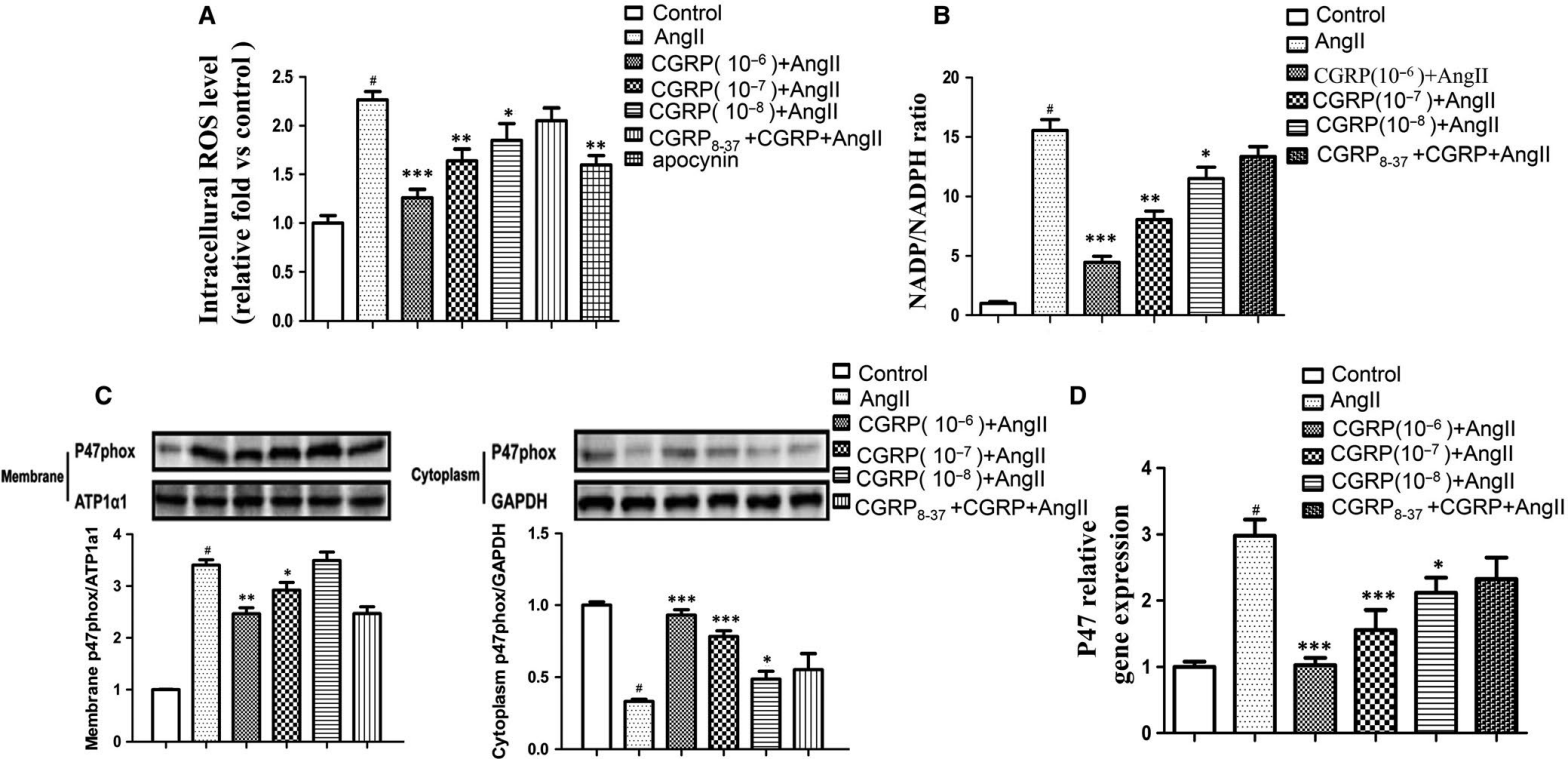
CGRP inhibited oxidative stress in Ang II‐induced VSMCs. Vascular smooth muscle cells (VSMCs), stimulated with CGRP (10^−6^‐10^−8^ mol/L) for 60 min, CGRP_8‐37_ (3 × 10^−5^ mol/L) for 30 min or apocynin (10^−6^ mol/L) for 2 h, were treated with Ang II (10^−7^ mol/L) for 30 min. A, Quantification of intracellular reactive oxygen species (ROS) levels. ROS levels were measured using DCFH‐DA as described in method. B, Quantitation of the intracellular NADP/NADPH ratio. C, Western blot analysis of membrane and cytoplasmic fractions of p47phox in VSMCs. D, Quantitative real‐time PCR analysis of mRNA levels of p47phox in VSMCs. Bar graphs show mean ± SEM values from three independent experiments. *P < .05, **P < .01 and ***P < .001 vs Ang II. ^#^ *P* < .05 vs control

In this study, we first examined the effects of CGRP on the oxidative stress stimulated by Ang II in VSMCs. CGRP significantly suppressed ROS production in Ang II‐stimulated VSMCs in a time‐dependent (Figure S1A) and dose‐dependent (Figure 1A) manner. It is well known that the Ang II‐stimulated activation of NADPH oxidase, especially the p47phox subunit, results in ROS generation in VSMCs. We therefore evaluated NADPH oxidase activation and found that CGRP decreased the intracellular NADP/NADPH ratio (Figure 1B). The p47phox amount was reduced in the cytoplasmic fraction and increased in the membrane fraction after Ang II stimulation, but these effects were reversed in the CGRP‐pre‐treated VSMCs (Figure 1C). Consistently, CGRP treatment significantly decreased the p47phox mRNA levels in Ang II‐induced VSMCs (Figure 1D). Additionally, CGRP inhibited ROS generation in VSMCs to almost the same level as that observed after treatment of the cells with an NADPH oxidase‐specific inhibitor, apocynin (Figure 1A). These data suggest that CGRP inhibited Ang II‐induced oxidative stress in VSMCs by inhibiting both NADPH oxidase activation and ROS production.

### 3.2 CGRP inhibited oxidative stress via receptors and the cAMP/PKA‐dependent pathway

**FIGURE 2:**
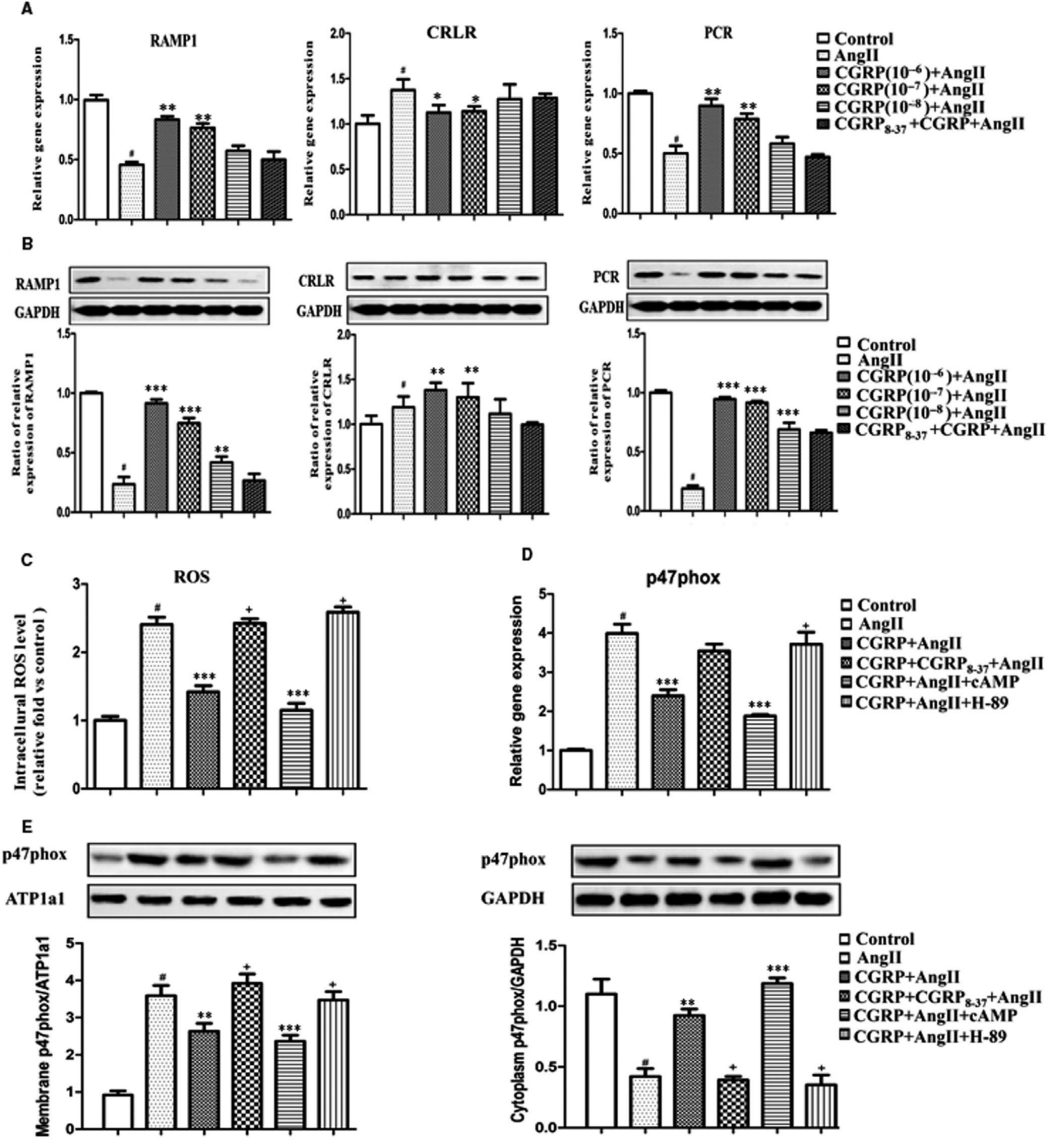
CGRP inhibited oxidative stress via receptors/ cAMP‐PKA‐dependent pathway. VSMCs, stimulated with CGRP (10^−6^‐10^−8^ mol/L) for 60 min or CGRP_8‐37_ (3 × 10^−5^ mol/L) for 30 min, were treated with Ang II (10^−7^ mol/L) for 30 min. In some experiments, dibutyl‐cAMP (10^−3^ mol/L) was applied for 60 min and H‐89 was applied 30 min before CGRP pre‐treatment. A, Quantitative real‐time PCR analysis of the mRNA levels of CRLR, RAMP1 and RCP in VSMCs. B, Western blot analyses of protein levels of CRLR, RAMP1 and RCP in VSMCs. C, Quantification of intracellular ROS levels in VSMCs after pre‐treatment with dibutyl‐cAMP or H‐89. D, Quantitative real‐time PCR analysis of mRNA levels of p47phox in VSMCs. E, Western blot analysis of membrane and cytoplasmic fractions of p47phox in VSMCs. Bar graphs show mean ± SEM values from three independent experiments. *P < .05, **P < .01 and ***P < .001 vs Ang II. ^#^ *P* < .05 vs control. ^+^ *P* < .05 vs CGRP + Ang II

CGRP exerts its biological effects by activating the cAMP/PKA‐dependent signalling pathway by binding to its receptors, the CRLR/RAMP1/RCP system. To identify the role of the CGRP receptor–dependent signalling pathway in inhibiting Ang II‐stimulated oxidative stress in VSMCs, we first examined the changes in CGRP receptors following Ang II induction. Both the mRNA and protein levels of CRLR increased in VSMCs after Ang II stimulation, whereas RAMP1 and RCP levels decreased significantly. However, these changes in the receptor levels were reversed by CGRP stimulation in a dose‐dependent manner (Figure 2A,B).

In VSMCs pre‐treated with a CGRP receptor antagonist (CGRP_8–37_), the CGRP‐mediated inhibition of Ang II‐induced oxidative stress was completely abolished, including the inhibition of p47phox activation and ROS production (Figure 1). We further examined the involvement of the cAMP/PKA‐dependent pathway in the CGRP inhibition of oxidative stress generation. The cAMP and PKA concentrations in Ang II‐stimulated VSMCs increased after CGRP stimulation, and the effect was again blocked by CGRP_8–37_ treatment (Figure S2A,B). Pre‐treatment of the VSMCs with dibutyl‐cAMP further promoted the inhibitory effect of CGRP on ROS generation, whereas pre‐treatment with H‐89 (a PKA inhibitor) completely abrogated the inhibitory effect (Figure 2C). We then examined the mRNA levels of p47phox, and its translocation in Ang II‐stimulated and CGRP‐treated cells, and found that dibutyl‐cAMP and H‐89 treatments had similar effects (Figure 2D,E).

### 3.3 CGRP attenuated Ang II‐induced Src/STAT3 activation in VSMCs

**FIGURE 3:**
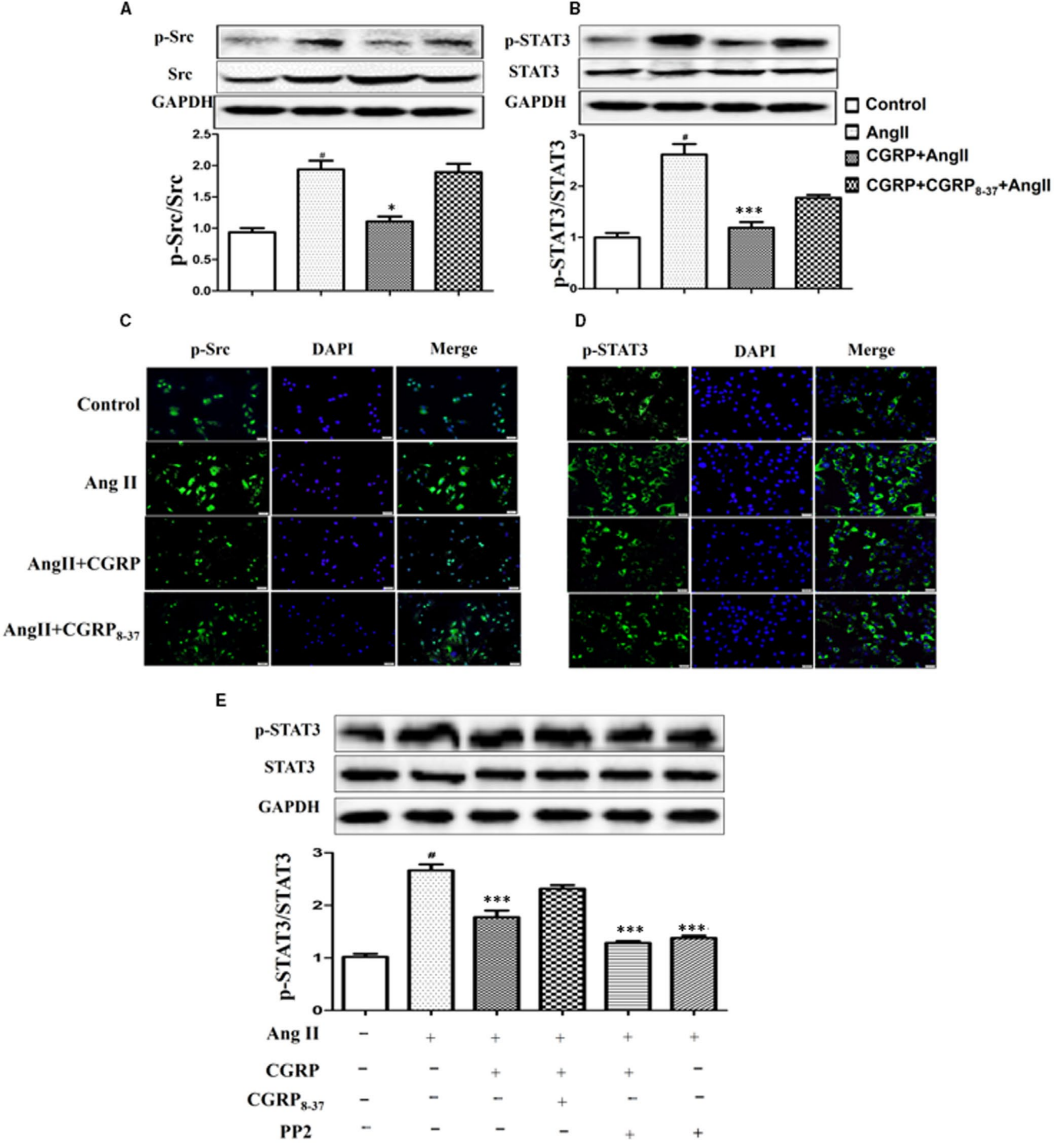
CGRP attenuated Ang II‐induced Src/STAT3 activation in VSMCs. VSMCs, stimulated with CGRP (10^−7^ mol/L) for 60 min or CGRP_8‐37_ (3 × 10^−5^ mol/L) for 30 min, were treated with Ang II (10^−7^ mol/L) for 30 min. A and B, Phosphorylation of Src and STAT3 was evaluated via Western blot analysis. C and D, The distribution and levels of p‐Src and p‐STAT3 were detected via immunofluorescence. E, VSMCs were treated with PP2 (10^−5^ mol/L, 20 min), and phosphorylation of STAT3 was evaluated via Western blot analysis. Bar graphs show mean ± SEM values from three independent experiments. *P < .05, **P < .01 and ***P < .001 vs Ang II. ^#^ *P* < .05 vs control

Both Src and STAT3 have been suggested to be involved in the regulation of Ang II‐induced VSMC responses. Therefore, the role of the Src/STAT3 signalling pathway was examined. As shown in Figure 3A,B, Ang II treatment enhanced the phosphorylation of both Src (Tyr416) and STAT3 (Tyr705), whereas CGRP treatment significantly attenuated the Ang II‐induced Src/STAT3 activation, an effect that was also verified by immunofluorescence assays (Figure 3C,D; Figure S3). Pre‐treatment of the cells with CGRP_8–37_ completely abolished the inhibitory effect of CGRP on Src/STAT3 activation (Figure 3A‐D).

The Src family of tyrosine kinases have been reported to mediate the phosphorylation of STAT3. We then examined the protein level of STAT3 in the Ang II or CGRP‐treated cells that had been pre‐treated with PP2, an Src inhibitor. We found out that phosphorylation of STAT3 induced by Ang II alone was suppressed when compared with that after PP2 pre‐treatment. The result indicated that Src was an upstream kinase candidate mediating Ang II‐induced STAT3 activation in VSMCs. Again, PP2 further strengthened the inhibitory effect of CGRP on STAT3 phosphorylation, where the level of STAT3 tended to be lower than the combination of Ang II and CGRP treatments. However, there was no significant difference (Figure 3E). The results showed that CGRP inhibition of Ang II‐induced STAT3 activation was associated with changes in the Src activity.

### 3.4 CGRP attenuated Ang II‐induced Src/STAT3 activation via the receptor/cAMP/PKA‐dependent pathway in VSMCs

**FIGURE 4:**
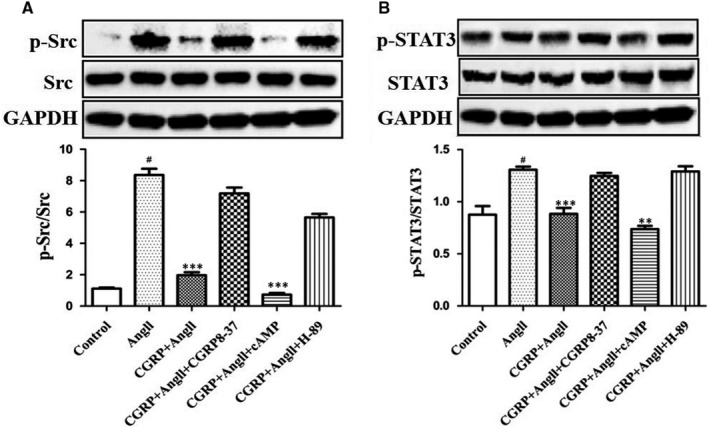
CGRP attenuated Ang II‐induced Src/STAT3 activation via receptor/cAMP‐PKA‐dependent pathway in VSMCs. VSMCs, stimulated with CGRP (10^−7^ mol/L) for 60 min or CGRP_8‐37_ (3 × 10^−5^ mol/L) for 30 min, were treated with Ang II (10^−7^ mol/L) for 30 min. In some experiments, dibutyl‐cAMP (10^−3^ mol/L) and H‐89 (10^−5^ mol/L) were applied 60 and 30 min before CGRP pre‐treatment, respectively. Phosphorylation of Src (A) and STAT3 (B) was evaluated via Western blot analysis. Bar graphs show mean ± SEM values from three independent experiments. *P < .05, **P < .01 and ***P < .001 vs Ang II. ^#^ *P* < .05 vs control

It is becoming clear that the Src and STAT3 pathways are potential effector pathways for G proteins and may play a role in G protein function. Therefore, we investigated whether CGRP attenuated Src/STAT3 activation through a G protein–coupled receptor (GPCR)–mediated signalling cascade, that is via the cAMP/PKA‐dependent pathway. Figure 4A,B shows the effects of dibutyl‐cAMP, H‐89 and CGRP_8–37_ treatment on Src and STAT3 phosphorylation. We found that dibutyl‐cAMP further promoted the inhibitory effect of CGRP on Src and STAT3 phosphorylation, whereas H‐89 treatment completely abolished this inhibitory effect.

### 3.5 The Src/STAT3 signalling pathway was involved in the antioxidative activity of CGRP

**FIGURE 5:**
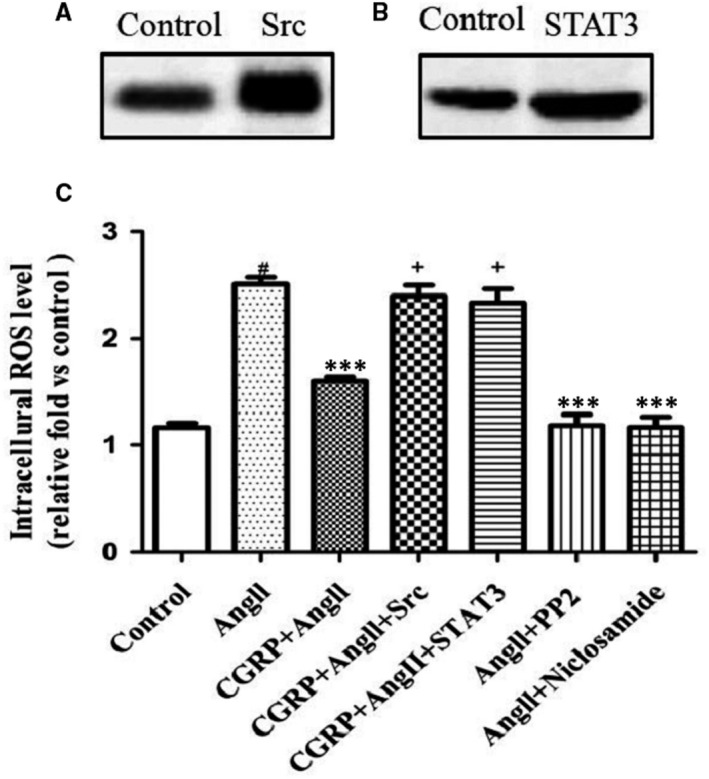
Src/STAT3 signalling pathway is involved in the antioxidant activity of CGRP. VSMCs, stimulated with CGRP (10^−7^ mol/L) for 60 min or CGRP_8‐37_ (3 × 10^−5^ mol/L) for 30 min, were treated with Ang II (10^−7^ mol/L) for 30 min. In some experiments, PP2 (10^−5^ mol/L) or niclosamide (10^−5^ mol/L) was applied 20 min before CGRP pre‐treatment. VSMCs were transfected with Src ORF cDNA clones (Src) or STAT3 ORF cDNA clones (STAT3) and incubated for 24 h. Increased Src (A) and STAT3 (B) protein levels in VSMCs were evaluated via Western blot analysis after transfection with Src or STAT3 ORF cDNA clones, respectively. (C), Quantification of intracellular ROS levels in VSMCs via DCFH‐DA. Src group, VSMCs were transfected with Src ORF cDNA clones; STAT3 group, VSMCs were transfected with STAT3 ORF cDNA clones; and control group, VSMCs were transfected with blank vector. Bar graphs show mean ± SEM values from three independent experiments. *P < .05, **P < .01 and ***P < .001 vs Ang II. ^#^ *P* < .05 vs control. ^+^ *P* < .05 vs CGRP + Ang II

Next, we investigated the role of Src and STAT3 in the inhibitory effect of CGRP on Ang II‐induced ROS generation by treating VSMCs with cDNA clones of the Src and STAT3 open reading frames (ORFs), the Src inhibitor PP2, or niclosamide, a selective STAT3 inhibitor. The transfection efficiency was confirmed via Western blot analysis for the Src and STAT3 proteins. As shown in Figure 5A,B, 24 hours after transfection, both Src and STAT3 expression levels increased significantly. In cells transfected with the Src or STAT3 ORF cDNA, CGRP treatment failed to inhibit high ROS generation. In contrast, PP2 and niclosamide significantly suppressed ROS generation induced by Ang II (Figure 5C).

### 3.6 CGRP suppressed the hypertrophy and hyperplasia of VSMCs in vitro and in vivo

**FIGURE 6:**
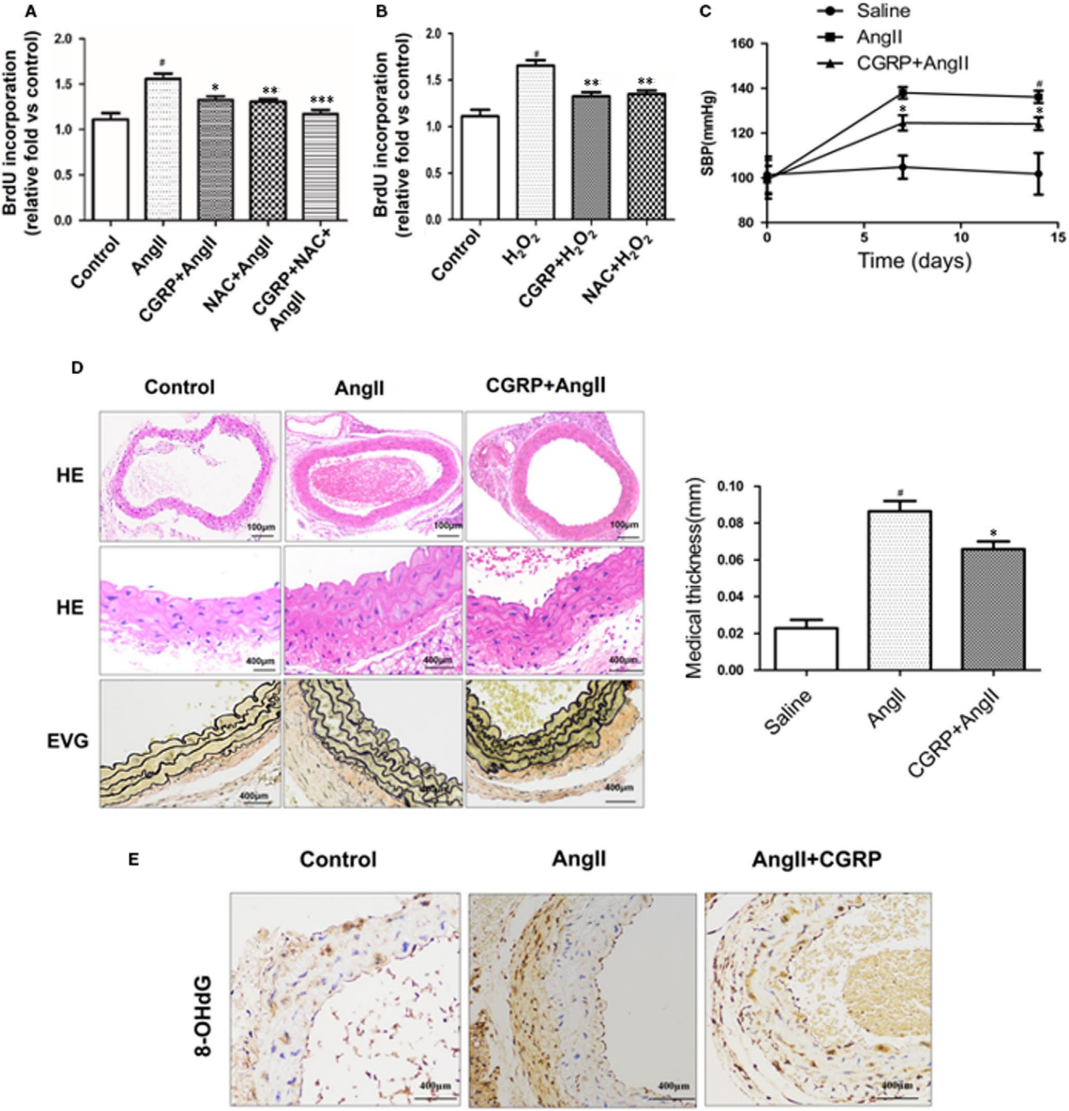
CGRP suppressed hypertrophy and hyperplasia of VSMCs in vitro and in vivo*.* VSMCs, stimulated with CGRP (10^−7^ mol/L) for 60 min or CGRP_8‐37_ (3 × 10^−5^ mol/L) for 30 min, were treated with Ang II (10^−7^ mol/L) for 30 min. In some experiments, NAC (10^−2^ mol/L) or H_2_O_2_ (10^−3^ mol/L) was applied 30 min before CGRP or Ang II pre‐treatment. A and B, The proliferation of VSMCs was analysed using BrdU assays. C, Systolic blood pressure (SBP) was measured by tail‐cuff plethysmography in three groups: control group (saline‐treated), Ang II (750 µg/kg/d, in saline)‐treated group and Ang II + CGRP (50 nmol/d, in saline)‐treated group (n = 5). D, The medial thickness of the abdominal aortas was assayed in the Ang II‐treated and Ang II + CGRP‐treated groups (n = 5). E, The 8‐OHdG immunohistochemistry of the abdominal aortas in the Ang II‐treated and Ang II + CGRP‐treated groups (n = 5). Bar graphs show mean ± SEM values from three independent experiments. *P < .05, **P < .05 vs Ang Ⅱ. ^#^ *P* < .05 vs control

To ascertain whether CGRP suppresses the vascular hypertrophy and hyperplasia of VSMCs by inhibiting oxidative stress, we first evaluated its inhibitory effect on the Ang II‐stimulated proliferation of VSMCs, using BrdU uptake as an index. As shown in Figure 6A, CGRP treatment significantly decreased Ang II‐stimulated proliferation of VSMCs to almost the same degree as that by the antioxidant, N‐acetylcysteine (NAC), which was used as a positive control. To further determine the association between the antiproliferative and antioxidative effects of CGRP, we evaluated the effect of CGRP on H_2_O_2_‐induced VSMC proliferation (Figure 6B). Both CGRP and NAC reduced H_2_O_2_‐induced proliferation of VSMCs to the same extent, suggesting that CGRP suppressed Ang II‐induced VSMC proliferation through antioxidative action.

Then, we infused mice with Ang II for 14 days to examine the effects of CGRP on Ang II‐induced vascular hypertrophy. As CGRP has been reported to lower blood pressure, we verified this effect in the Ang II‐infused mice. As expected, Ang II infusion increased the systolic blood pressure (SBP) of the mice, whereas hypertension was significantly reversed in CGRP‐treated mice (Figure 6C). HE and EVG staining showed that CGRP treatment significantly inhibited Ang II‐induced thickening of the abdominal aortas (Figure 6D). In addition, the immunostaining assay for 8‐OHdG, a marker for ROS‐induced DNA damage, showed that CGRP treatment had decreased the level of ROS damage induced by Ang II relative to that in the untreated mice (Figure 6E). Taken together, these in vitro and in vivo results suggest that CGRP may play key roles in the Ang II‐induced hypertrophy and hyperplasia of VSMCs by suppressing oxidative stress.

## 4 DISCUSSION

The important findings of this study are as follows: (a) CGRP suppressed NADPH oxidase‐generated ROS in Ang II‐induced VSMCs, and the antioxidative effect of CGRP might be mediated by the receptor/cAMP/PKA‐dependent signalling pathway activation; (b) CGRP inhibited the Src and STAT3 activation stimulated by Ang II via the receptor/cAMP/PKA‐dependent signalling pathway in VSMCs; (c) the Src‐dependent STAT‐3 signalling pathway was found to be involved in the antioxidative activity of CGRP; and (d) CGRP inhibited the hypertrophy and hyperplasia of VSMCs via its antioxidative effect both in vitro and in vivo.

CGRP has been reported to elicit protective effects against cell injuries in several disease models, where the mechanisms underlying its protective effects differed depending on the cell types and experimental conditions.^11^, ^20^, ^21^, ^22^, ^23^ We had previously reported that CGRP deficiency led to enhanced neointima formation after vascular injury, where the cells also showed a higher degree of oxidative stress.^10^ In the present study, we found that CGRP inhibited Ang II‐induced oxidative stress, both in vitro and in vivo, including the inhibition of NADPH oxidase activation, p47phox activation and ROS production. As NADPH oxidase is an important enzyme for ROS production, we determined whether CGRP reduced ROS production by inhibiting NADPH oxidase, using apocynin (an NADPH oxidase inhibitor) as a positive control for the in vivo study. We found that both CGRP and apocynin reduced Ang II‐stimulated ROS generation to a similar degree. Thus, CGRP may reduce the ROS generated in Ang II‐induced VSMCs by inhibiting NADPH oxidase directly.

CGRP is involved in the regulation of the cardiovascular system via its receptors. For instance, in an animal model of Ang II‐induced hypertension, RAMP1 transgenic mice were shown to be protected from increases in their SBP.^24^ However, the CGRP and/or CGRP receptor expression profile varies with the state and stage of the disease, including that in the Ang II‐induced model.^25^, ^26^ In addition, the change in CGRP receptor expression was suggested to further amplify the responses to CGRP, especially in the hypertension model.^27^ In our study, we found that RAMP1 and RCP were down‐regulated and CRLR was up‐regulated in Ang II‐induced VSMCs, and CGRP reversed these Ang II effects. Moreover, the CGRP‐stimulated normalization of the expression of these receptors was consistent with its concentration‐dependent inhibitory effect on Ang II‐induced oxidative stress. In addition, treatment of the VSMCs with CGRP_8–37_, an inhibitor of the CGRP receptors, suppressed the effects of CGRP. In view of these results, it appears that CGRP may act as a regulatory autocrine or paracrine factor via its receptors to exert its functions in Ang II‐induced VSMCs.

The intracellular signalling mediated by CGRP and its receptors is highly complex. The cAMP/PKA‐dependent pathway is the primary signalling pathway involved in the CGRP effect on the vasculature, especially in VSMCs.^28^ We observed that CGRP increased the intracellular cAMP content and PKA activity in Ang II‐stimulated VSMCs. In order to investigate whether CGRP receptor–mediated cAMP/PKA‐dependent signalling is responsible for the antioxidative effect of CGRP, VSMCs were incubated with CGRP_8–37_, dibutyl‐cAMP or H‐89. CGRP_8–37_ and H‐89 abolished the inhibitory effect of CGRP on ROS production and p47phox activation, respectively, whereas dibutyl‐cAMP further enhanced the inhibitory effect. These data indicated that the CGRP receptor/cAMP/PKA‐dependent pathway was involved in the CGRP‐mediated down‐regulation of ROS generation by the Ang II‐stimulated NADPH oxidase.

Activation of the CGRP receptors can cause the activation of multiple signalling pathways and the subsequent recruitment of several downstream effectors.^29^ GPCRs are capable of activating Src and STAT3, and modulating several physiological processes, such as cell proliferation and transformation.^30^ Src can be activated by Ang II and play important roles in the signalling events associated with VSMC contraction and proliferation.^31^, ^32^ In addition, Src is involved in the activation of STAT, which also regulates VSMC proliferation and migration.^17^, ^33^ We propose here, for the first time, that Src was an upstream kinase candidate mediating Ang II‐induced STAT3 activation in VSMCs, as the inhibition of Src by PP2 blocked STAT3 activation. Meanwhile, treatment with CGRP significantly suppressed Ang II‐induced activation of Src and STAT3, and pre‐treatment with CGRP_8–37_ or H‐89 abolished the inhibitory effect of CGRP. Our in vivo data showed that the Src/STAT3 signalling pathway was modulated by the CGRP receptor/cAMP/PKA‐dependent pathway in response to Ang II in VSMCs, demonstrating the role of Src/STAT3 signalling in the CGRP‐associated antioxidative activity.

Numerous studies have reported that Src or STAT3 is involved in the regulation of oxidative stress.^18^, ^34^, ^35^ Similarly, our results showed that the inhibition of Src or STAT3 significantly inhibited ROS generation induced by Ang II. In addition, Src or STAT3 overexpression reversed the inhibitory effect of CGRP on Ang II‐induced ROS generation. In view of these results, it can be speculated that the Src/STAT3 signalling pathway, in association with the activation of CGRP receptors, contributes to the CGRP‐mediated effects of regulating Ang II‐induced oxidative stress in VSMCs.

Ang II‐mediated vascular hypertrophy is implicated in VSMC proliferation, where ROS has been shown to play a crucial role. In the current study, we showed that CGRP suppressed Ang II‐induced VSMC proliferation in vitro and vascular hypertrophy in vivo, accompanied by a decrease in ROS production. The ROS‐lowering effect of CGRP was similar to that of NAC (a ROS scavenger), which also inhibited Ang II‐induced VSMC proliferation. As the most important ROS in pathological conditions are superoxide (O^2−^) and H_2_O_2_,^36^ we induce VSMC proliferation, using H_2_O_2_, and confirmed that the antiproliferative effects of CGRP were mediated by directly reducing ROS generation. Both CGRP and NAC reduced H_2_O_2_‐induced VSMC proliferation, suggesting that CGRP activity is redox‐sensitive. Our in vivo and in vitro data demonstrated that targeting ROS generation may be a critical mechanism by which CGRP mediates its antiproliferative effects on Ang II‐stimulated VSMCs. Thus, CGRP alleviates the Ang II‐induced hypertrophy and hyperplasia of VSMCs via the inhibition of ROS generation.

In summary, our results demonstrate that CGRP exhibits its antioxidative effect by blocking the Src/STAT3 signalling pathway, which is associated with the hypertrophy and hyperplasia of VSMCs induced by Ang II. Moreover, the current study provides molecular evidence in support of previous reports on the important role of CGRP in protecting against Ang II‐induced oxidative stress in VSMCs during the development of hypertension.

## CONFLICT OF INTEREST

The authors declare that there are no conflicts of interest.

## AUTHOR CONTRIBUTION

Lei Yang, Wen‐xuan Liu, Hong‐min Luo and Xia Wu provided theoretical guidance and designed experiments. Lei Yang, Hong‐min Luo, Xia Wu, Xian Xian, Lu‐yao Wang, Liang‐yu Zhu and Hong‐yu Sun performed experiments and analysed results. Wen‐xuan Liu, Hong‐min Luo and Xia Wu wrote the manuscript. All authors revised the manuscript and contributed substantially to this research.

## Supporting information

Fig S1

Fig S2

Fig S3

## Data Availability

The data used to support our findings of this study are available on request from the corresponding author.
