# Supplementary figures and images for: Calcitonin gene‐related peptide inhibits angiotensin II‐induced NADPH oxidase‐dependent ROS via the Src/STAT3 signalling pathway
Source: J Cell Mol Med. 2020 May 5;24(11):6426–37. doi: 10.1111/jcmm.15288 (PMC7294141; doi:10.1111/jcmm.15288)

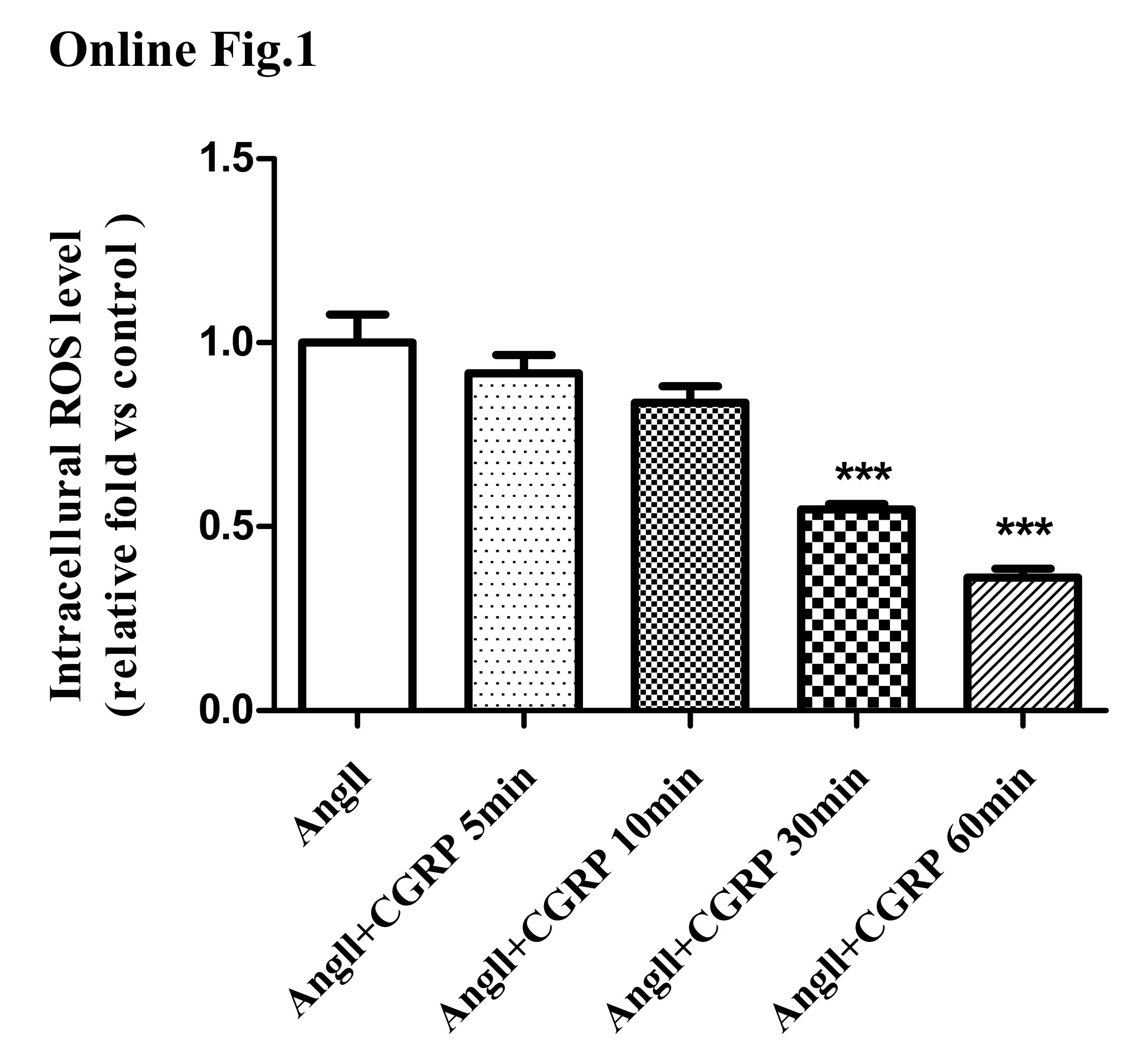

Supplement: Supplementary file 1 — Fig S1 [file JCMM-24-6426-s001.tif]

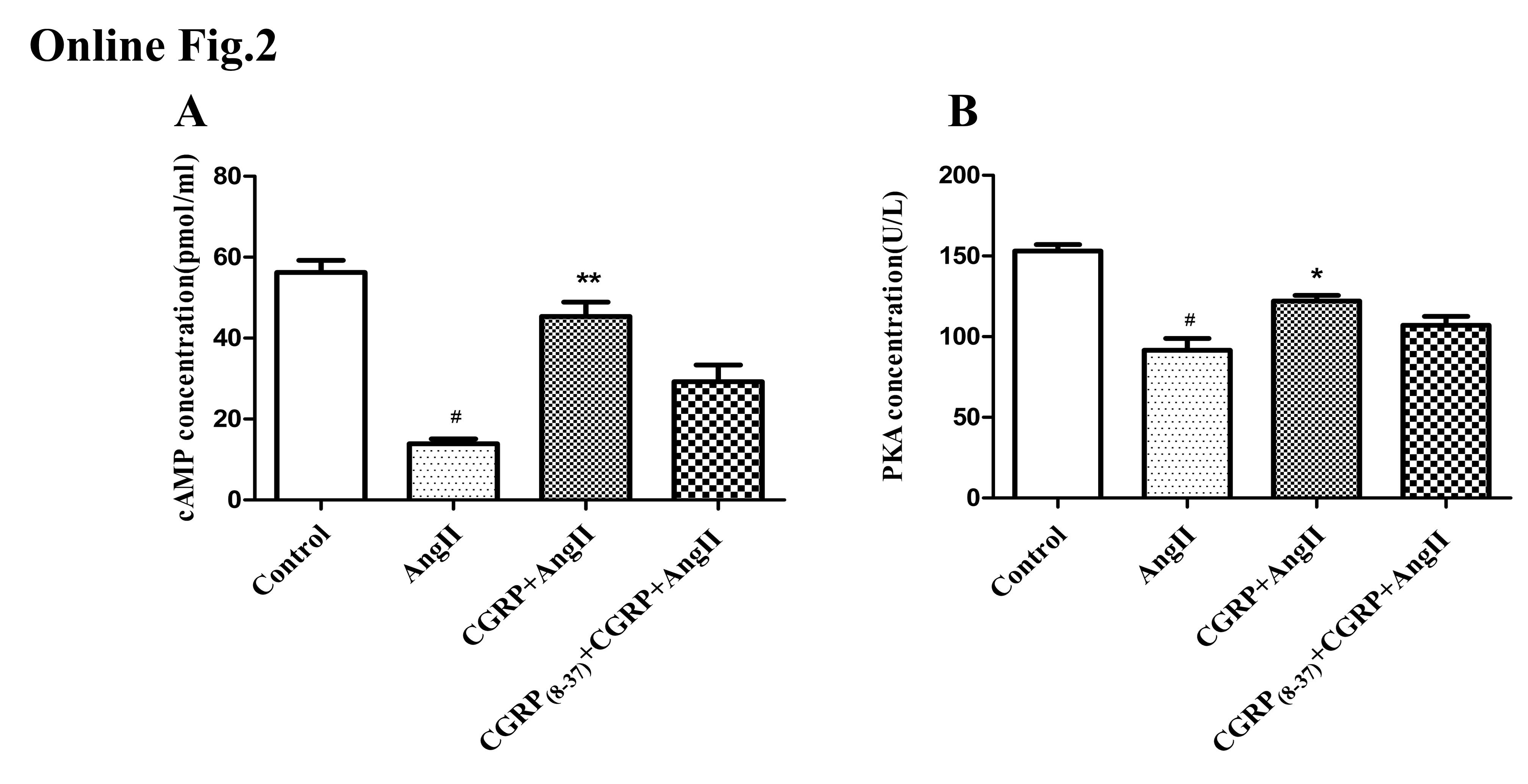

Supplement: Supplementary file 2 — Fig S2 [file JCMM-24-6426-s002.tif]

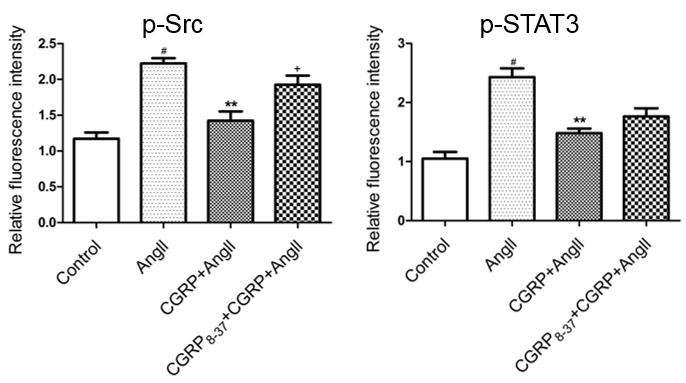

Supplement: Supplementary file 3 — Fig S3 [file JCMM-24-6426-s003.tif]
